# Supplementary material for: Effect of Amino Acids on Folates Accumulation in Wheat Seedlings during Germination under Red Light Radiation
Source: Molecules. 2022 Oct 13;27(20):6868. doi: 10.3390/molecules27206868 (PMC9607551; doi:10.3390/molecules27206868)
Supplement: Supplementary file 1 [file molecules-27-06868-s001.zip › molecules-1928733-supplementary.pdf]

Supplementary Materials

Effect of Amino Acids on Folates Accumulation in Wheat Seedlings during Germination under Red Light Radiation

Chong Xie, Pei Wang, Jianwei Chang, Qiaoe Wang, Yongbin Han and Runqiang Yang*

Table S1. Correlation between the production of folates with the gene expression of four enzymes.

	GTPCH1	*ADCS*	*HPPK/DHPS*	*FPGS*
ADCS	0.61*			
HPPK/DHPS	0.45*	−0.27		
FPGS	0.63**	0.14	0.38	
Folates	0.80**	0.63**	0.29	0.62**

GTPCH1: guanosine triphosphate cyclohydrolase I, *ADCS*: aminodeoxychoris-mate synthase, *HPPK/DHPS*: 6-hydroxymethyldihydropterin pyrophosphoki-nase/dihydropteroate synthase, *FPGS*: folylpolyglutamate synthetase. *$p < 0.05$; **$p < 0.01$.

Table S2. Parameters for determination of folates and precursors standards.

Folate	Precursor ion (*m/z*)	Product ion (*m/z*)	DP (V)	EP(V)	CE (V)	CXP(V)
THF	446.2	299.3	80	10	25	10
	446.2	166.3	80	10	59	10
5-CH_3-THF	460.3	313.2	70	10	27	10
	460.3	194.2	60	10	47	10
5-CHO-THF	474.2	299.3	61	10	43	10
	474.2	166.3	120	10	54	10
10-CHO-FA	470.1	295.3	65	10	35	10
	470.1	176.2	86	10	59	10
*p*ABA	138.0	77.0	80	10	30	10
	138.0	94.0	80	10	19	10
Pterin	194.0	176.0	60	10	23	10
	194.0	106.0	60	10	39	10

Note: DP: declustering potential; EP: entrance potential; CE: collision energy; CXP: collision cell exit potential; V: volt.

Table S3. Sequences of primers used in this study.

Gene	Primer name	Primer sequences(5′→3′)	Gene Bank Access No.
GTPCH1	Sense	GAGGACCCCCTAAGGAAAGA	EF095153.1
	Ant-sense	CTGGGCACAAAATGGCAA	
ADCS	Sense	ATGCTTGCAGAGAGAAACAGC	EF208804.1
	Ant-sense	CCGCCCTTACCACCCATAAA	
HPPK/DHPS	Sense	TCTTGGAAGCAATGTGGGGG	EF208803.1
	Ant-sense	GGCTGGTCGGTCACATACTC	
FPGS	Sense	CCAAGCAGTGTGGGAGAAAT	EF208805.1
	Ant-sense	GGGAGCCAGTAACAAGGACC	
